# Disability Status as an Antecedent to Chronic Conditions: National Health Interview Survey, 2006–2012

**DOI:** 10.5888/pcd11.130251

**Published:** 2014-01-30

**Authors:** Alicia Dixon-Ibarra, Willi Horner-Johnson

**Affiliations:** Author Affiliation: Willi Horner-Johnson, PhD, Oregon Health and Science University, Portland, Oregon.

## Abstract

**Introduction:**

A strong relationship exists between disability and poor health. This relationship could exist as a result of disabilities emerging from chronic conditions; conversely, people with disabilities may be at increased risk of developing chronic conditions. Studying health in relation to age of disability onset can illuminate the extent to which disability may be a risk factor for future poor health.

**Methods:**

We used data from the 2006–2012 National Health Interview Survey and conducted weighted logistic regression analyses to compare chronic conditions in adults with lifelong disabilities (n = 2,619) and adults with no limitations (n = 122,395).

**Results:**

After adjusting for sociodemographic differences, adults with lifelong disabilities had increased odds of having the following chronic conditions compared with adults with no limitations: coronary heart disease (adjusted odds ratio [AOR] = 2.92; 95% confidence interval [CI], 2.33–3.66) cancer (AOR = 1.61; 95% CI, 1.34–1.94), diabetes (AOR = 2.57; 95% CI, 2.10–3.15), obesity (AOR = 1.81; 95% CI, 1.63–2.01), and hypertension (AOR = 2.18; 95% CI, 1.94–2.45). Subpopulations of people with lifelong disabilities (ie, physical, mental, intellectual/developmental, and sensory) experienced similar increased odds for chronic conditions compared with people with no limitations.

**Conclusion:**

Adults with lifelong disabilities were more likely to have chronic conditions than adults with no limitations, indicating that disability likely increases risk of developing poor health. This distinction is critical in understanding how to prevent health risks for people with disabilities. Health promotion efforts that target people living with a disability are needed.

## Introduction

Healthy People 2020 calls for increasing inclusion of people with disabilities in US health promotion efforts (1). Data indicate that chronic conditions are prevalent among people with disabilities (2–6); however, research that elucidates temporal relationships between disability and health outcomes is limited. Considerable research has addressed disability as a consequence of chronic conditions (7–12). Only a few studies have examined health conditions developed after disability onset, and these have focused on people with mobility limitations, especially spinal cord injuries (13–16).

Whether disability is a result of or a risk factor for chronic conditions has implications for health promotion and disease prevention efforts (17). If disability is viewed primarily as resulting from chronic conditions, then health promotion logically concentrates on people without disabilities and emphasizes prevention and control of conditions that could lead to disability. If, however, people who have disabilities are at increased risk for developing chronic conditions, then these populations are indeed an important target for health promotion efforts, as indicated by Healthy People 2020.

National longitudinal data are lacking on chronic conditions that manifest after disability onset. However, some cross-sectional data allow investigation of temporal relationships between disability onset and health. For example, Jamoom and colleagues used data on age of disability onset in the Behavioral Risk Factor Surveillance System to compare the health status of people with developmentally acquired disabilities to that of those with disabilities acquired later in life (18). Similar approaches can be used with data from the National Health Interview Survey (NHIS), which includes questions about how long respondents have had various limitations. The purpose of this study was to use NHIS data to determine whether chronic conditions are more prevalent in people with lifelong disability compared with people without limitations.

## Methods

### Data source

The NHIS is an annual cross-sectional survey sponsored by the National Center for Health Statistics (NCHS). NHIS consists of groups of surveys that collect data on households and individuals. It collects health and health service utilization information on civilian households and noninstitutionalized populations in the United States. In 2012, the NHIS sampled approximately 46,000 households and 116,000 household members with 201 primary sampling units, which is typical of most data collection years (19). The data for this study were obtained from the Sample Adult file, which contains health-related data from 1 randomly selected adult (aged 18 years or older) in each household. The information in the Sample Adult file is self-reported unless the respondent selected is incapable of answering, in which case a proxy in the household is selected to supply respondent information (19).

For our analyses, we pooled Sample Adult files from 2006 through 2012. The estimate from the pooled analysis was interpreted as an average over the time interval of data. The sampling weight was adjusted using the NCHS recommendation to divide the sample weight in the pooled data set by the number of combined years (ie, sample weight divided by 7) (19). Variables in the pooled data set were checked for changes across survey years.

### Measures

NHIS collected data on the duration of limitation from people with disabilities. We used these data and current age to determine the age of limitation onset to specify people with lifelong disabilities. NHIS also collects data on chronic conditions including coronary heart disease, cancer, diabetes, hypertension, and obesity. These were selected as outcomes in this analysis on the basis of data that indicate that heart disease, cancer, and diabetes are among the most common, costly, and preventable health problems in the United States (20). Hypertension and obesity were included as conditions contributing to onset of 1 or more of the other conditions.

#### Lifelong disability

Respondents were asked in preliminary questions if they had physical, mental, or emotional problems resulting in a limitation or inability to work; difficulty walking without special equipment; limitations in activities of daily living (ie, bathing, dressing, getting in/out of bed, using the toilet, eating, or getting around the home); and limitations in instrumental activities of daily living (ie, household chores, doing necessary business, shopping, or getting around) or any other activity. They were further asked about the condition or health problem associated with their limitation and the amount of time they had the condition. The following 18 fixed condition categories were provided to the sample adult to choose from: “vision/problem seeing,” “hearing problem,” “arthritis/rheumatism,” “back or neck problem,” “fracture, bone/joint injury,” “other injury,” “heart problem,” “stroke problem,” “hypertension/high blood pressure,” “diabetes,” “lung/breathing problem (eg, asthma, emphysema),” “cancer,” “birth defect,” “intellectual disability, also known as mental retardation,” “other developmental problem (eg, cerebral palsy),” “senility,” “depression/anxiety/emotional problem,” and “weight problem.” If the sample adult was limited by conditions not specified, the interviewer coded responses into 1 of 17 additional categories (not read aloud to respondents), as applicable (19).

For these analyses, we subtracted the reported number of years with a limitation from the age at the time of interview to obtain age of disability onset. We then used this information to create a lifelong disability variable. The case definition for lifelong disability included people who identified a limitation with onset before age 6 and excluded people with “lifelong” old age–related problems (as coded in NHIS data), senility, surgical after-effects, drug and alcohol problems, lung and breathing problems, pregnancy conditions, and the category of “other,” which included a diverse group of responses not specified in the 18 fixed conditions. The rationale for defining lifelong disability as having onset before age 6 was that, historically, many children have not had their disabilities diagnosed until starting school (typically at age 5). Our exclusion criteria also involved removing people who identified lifelong conditions similar to our outcomes of interest (ie, heart problems, cancer, diabetes, weight problems, and stroke), so our analyses could focus on chronic conditions developed subsequent to disability.

To examine subpopulations with lifelong disabilities, we established the following case definitions for analyses. The case definition for physical disability included people who identified a physical limitation (ie, arthritis or rheumatism, back or neck problem, fracture, bone/joint injury, birth defect, missing limb, or musculoskeletal problem). Mental disability included people who identified a limitation due to mental health (ie, depression, anxiety, emotional problems, or another mental problem). Intellectual and developmental disability included people who identified an intellectual or other developmental limitation. People with sensory disabilities identified having a limitation with vision or hearing. Categories were not mutually exclusive and could include people who had 1 or more other types of limitations. All subgroups had onset before age 6.

#### Chronic conditions

Coronary heart disease, cancer, diabetes, and hypertension were established as dichotomous variables on the basis of responses to interview questions asking, “Have you ever been told by a doctor or other health professional that you had [coronary heart disease, cancer, diabetes, or hypertension].” Respondents who answered yes to a given condition were coded as having that condition. Obesity was derived from body mass index (BMI, kg/m^2^), which was calculated on the basis of self-reported weight and height. We coded respondents as obese if they had a BMI of 30 or more.

### Data analysis

Weighted descriptive statistics, representing the US adult noninstitutionalized population aged 18 years or older, were calculated. We conducted *t* tests and χ^2^ analyses to identify significant differences in sociodemographics and chronic conditions between people with lifelong disabilities and people without limitations. Odds ratios (ORs), adjusted ORs (AORs), and 95% confidence intervals (CIs) were calculated using separate binary logistic regression models for each chronic condition (ie, coronary heart disease, cancer, diabetes, obesity, and hypertension) while controlling for sociodemographic variables (age, sex, race/ethnicity, marital status, and working status). To examine subpopulations of people with lifelong disabilities and their relationship to chronic conditions, we conducted separate analyses for specific limitation groups: physical disability, mental disability, intellectual/developmental disabilities, and sensory disabilities. Given the role that age plays in the development of chronic disease, we additionally stratified our analysis into policy-relevant age categories of 18 to 44 years, 45 to 64 years, 65 to 79 years, and age 80 or older. STATA version 11 (StataCorp LP, College Station, Texas) statistical software was used for all analyses. Taylor series linearization method was used for variance estimation to accommodate the NHIS’s complex, multistage sampling design. The NCHS recommendation to maintain the full data file while conducting subpopulation analyses was performed by adding SUBPOP to the SVY statement in STATA.

## Results

A total of 2,619 respondents were classified as having a lifelong disability (Table 1), which yielded a population estimate of 5,563,362 adults with lifelong disabilities across the United States. People with lifelong disabilities differed significantly from the comparison group on all sociodemographic variables, and significantly higher proportions of people with lifelong disabilities reported each of the chronic conditions (Table 2).

**Table 1:** Sample Size by Survey Year, National Health Interview Survey, 2006–2012^a^

Year	Lifelong Disability					No Limitations
	Any^b^	Physical	Mental	Intellectual/Developmental	Sensory	
2006	277	116	87	33	27	16,101
2007	278	120	92	29	30	15,638
2008	276	110	89	24	30	14,191
2009	376	148	118	44	38	17,687
2010	392	149	141	53	46	17,007
2011	484	188	165	67	46	20,270
2012	536	211	189	75	53	21,501
Total	2,619	1,042	881	325	270	122,395

a Data source: National Health Interview Survey 2006–2012.

b Includes any limitation identified as having age of onset ≤6 years (not all are categorized into 1 of the listed disability types).

**Table 2:** Respondent Characteristics, People With Lifelong Disabilities and No Limitation, National Health Interview Survey, 2006–2012

Characteristic^a^	Lifelong Disability (n = 2,619)	No Limitation (n = 122,395)
**Age, mean (SE)^b^**	43.2 (0.5)	41.4 (0.1)
**Sex^b^**		
Male	47.1 (1.2)	51.8 (0.2)
Female	52.9 (1.2)	48.2 (0.2)
**Race/ethnicity^b^**		
Non-Hispanic white	73.9 (1.6)	65.2 (0.3)
Black	10.2 (0.8)	11.6 (0.2)
Hispanic	9.9 (0.7)	15.2 (0.2)
Other	6.1 (0.5)	7.9 (0.2)
**Marital status^b^**		
Married	34.5 (1.2)	55.1 (0.3)
Divorced	10.4 (0.6)	7.6 (0.1)
Widow	5.1 (0.4)	2.9 (0.1)
Separated	2.9 (0.4)	1.9 (0.04)
Never married	39.8 (1.3)	25.2 (0.3)
Part of unmarried couple	7.3 (0.7)	7.4 (0.1)
**Work status in past year^b^**		
Not working	51.3 (1.3)	20.2 (0.2)
Working	48.7 (1.3)	79.8 (0.2)
**Chronic condition^b^**		
Coronary heart disease	5.4 (0.5)	1.8 (0.1)
Cancer	8.8 (0.6)	4.8 (0.1)
Diabetes	10.5 (0.9)	4.4 (0.1)
Obesity	34.2 (1.1)	24.7 (0.2)
Hypertension	33.1 (1.1)	19.1 (0.2)

Abbreviation: SE, standard error.

a Values are expressed as % (SE) unless otherwise indicated. Percentages and standard errors are based on weighted data to represent the civilian noninstitutionalized US population. Because of nonresponse, percentage may not add up to 100. Data Source: National Health Interview Survey 2006–2012.

b *P* < .001.

After controlling for age, sex, race/ethnicity, marital status, and working status, people with lifelong disabilities had significantly higher odds of all chronic conditions compared with people without limitations. The largest effect was for coronary heart disease (AOR = 2.92; 95% CI, 2.33–3.66) (Table 3).

**Table 3:** Multivariate Analyses of Chronic Conditions by Lifelong Disability, National Health Interview Survey, 2006–2012^a^

Lifelong Disability		CHD	Cancer	Diabetes	Obesity	Hypertension
All lifelong disabilities (n = 2,619)	Crude OR	3.16 (2.59–3.85)	1.93 (1.64–2.28)	2.54 (2.11–3.05)	1.58 (1.43–1.75)	2.09 (1.89–2.31)
	AOR	2.92 (2.33–3.66)	1.61 (1.34–1.94)	2.57 (2.10–3.15)	1.81 (1.63–2.01)	2.18 (1.94–2.45)
**Disability type**						
Physical (n = 1,042)	Crude OR	3.42 (2.49–4.70)	2.13 (1.83–3.04)	2.52 (2.01–3.18)	1.73 (1.45–2.07)	2.35 (2.02–2.73)
	AOR	2.62 (1.83–3.75)	1.62 (1.22–2.15)	2.24 (1.75–2.87)	1.95 (1.62–2.35)	2.12 (1.78–2.52)
Mental (n = 881)	Crude OR	1.81 (1.18–2.78)	1.33 (0.96–1.83)	2.04 (1.33–3.14)	1.63 (1.37–1.93)	1.76 (1.46–2.12)
	AOR	2.48 (1.51–4.08)	1.56 (1.08–2.26)	2.81 (1.74–4.52)	1.88 (1.58–2.23)	2.45 (2.00–3.01)
Intellectual/ developmental (n = 325)	Crude OR	1.29 (0.46–3.59)	0.49 (0.24–1.01)	1.36 (0.79–2.34)	1.82 (1.34–2.47)	1.22 (0.88–1.69)
	AOR	2.66 (0.86–8.17)	0.93 (0.42–2.04)	2.34 (1.30–4.22)	2.30 (1.68–3.14)	2.11 (1.49–2.98)
Sensory (n = 270)	Crude OR	7.34 (4.64–11.59)	3.35 (2.25–4.98)	4.70 (3.14–7.04)	1.73 (1.28–2.33)	2.91 (2.17–3.91)
	AOR	5.07 (2.99–8.60)	2.17 (1.36–3.45)	3.69 (2.32–5.89)	2.09 (1.54–2.83)	2.29 (1.59–3.28)
**Age group**						
18–44 y (n = 1,218)	Crude OR	5.42 (3.33–8.81)	3.00 (2.26–3.99)	2.11 (1.57–2.84)	1.55 (1.38–1.75)	2.23 (1.93-2.58)
	AOR	4.51 (2.69–7.56)	2.65 (1.97–3.56)	2.03 (1.50–2.76)	1.65 (1.46–1.87)	2.28 (1.96–2.65)
45–64 y (n = 950)	Crude OR	3.45 (2.68–4.44)	1.69 (1.37–2.09)	2.39 (2.00–2.86)	1.71 (1.50–1.96)	2.27 (1.99-2.58)
	AOR	3.46 (2.65–4.52)	1.51 (1.21–1.87)	2.34 (1.94–2.83)	1.78 (1.55–2.04)	2.26 (1.97–2.58)
65–79 y (n = 324)	Crude OR	2.52 (1.89–3.37)	1.64 (1.27–2.12)	2.06 (1.59–2.66)	2.57 (2.04–3.23)	1.58 (1.26-1.98)
	AOR	3.06 (2.26–4.16)	1.64 (1.26–2.14)	2.31 (1.78–3.01)	2.63 (2.08–3.33)	1.64 (1.30–2.07)
≥80 y (n = 127)	Crude OR	1.51 (0.92–2.48)	1.05 (0.69–1.59)	1.77 (1.11–2.83)	1.45 (0.87–2.40)	1.71 (1.17-2.49)
	AOR	1.81 (1.09–3.02)	1.05 (0.68–1.62)	1.95 (1.19–3.17)	1.63 (0.97–2.72)	1.61 (1.10–2.35)

Abbreviations: CHD, coronary heart disease; OR, odds ratio; AOR, adjusted odds ratio.

a Adults with no limitation served as the reference for all comparisons. Adjusted for age, sex, race/ethnicity, marital status, and working status.

In general, the results of weighted crude and adjusted logistic regression demonstrated similar findings across subpopulations of people with lifelong disabilities, with increased odds of chronic conditions in each group (Table 3), compared with people with no limitations. People with physical disabilities, mental disabilities, and sensory impairments had increased odds for all conditions (coronary heart disease, cancer, diabetes, hypertension, and obesity). People with intellectual/developmental disabilities had increased odds for all specified chronic conditions except for coronary heart disease and cancer.

Results of the age-stratified analyses demonstrated that, regardless of disability status, chronic conditions increased with age. As the prevalence of chronic conditions increased in the nondisabled group in the higher age categories, the odds ratios for people with disabilities decreased in those categories (Table 3). However, nearly all of the differences between those with lifelong disabilities and no limitations were still significant in the older age categories.

## Discussion

Our findings can increase understanding of the temporal relationship between disability and poor health. Although public health efforts emphasize the need to address chronic conditions to prevent future disability, less attention has been paid to potential risk for health problems among those who already have a disability (17). Research that has investigated the latter question is primarily cross-sectional and based on chronic conditions and disability status at time of interview, precluding the ability to disentangle the reciprocal relationship between disability and health.

Although one previous study found a longitudinal relationship between disability status and development of new health problems (13), the initial health status of the persons with disabilities and the duration of their disability were less clear. A unique aspect of our study is its use of data on past exposure (early disability onset) to examine associations with adult chronic conditions. By focusing on people with lifelong disability, we excluded those with later-onset disabilities that may have occurred pursuant to chronic conditions. We also excluded people who reported limitations from an early age that were related to any of our outcomes. Thus, to the extent possible with the available data, we in essence compared people with and without lifelong limitations starting with a “clean slate” from a health standpoint.

Future directions in investigating health risks for people with disabilities should include examining the temporal relationship of disability and the onset of chronic conditions, especially in cohort analyses. For example, adding disability indicator questions to large-scale longitudinal cohort studies, such as the Framingham Heart Study or the Nurses’ Health Study, could facilitate more detailed analyses of the temporal relationship between disability and chronic conditions, regardless of whether disabilities are lifelong or acquired later in life. Future research should also separately examine the health trajectories of people with more than 1 type of disability. Moreover, examining determinants of future health risk should also be a key area of inquiry, particularly exploring environmental factors for people with disabilities (eg, accessibility), social determinants of health, and personal factors. Exploring social and environmental aspects of disability is important in understanding a person’s functioning and future health risks and in creating appropriate interventions. Such an approach is consistent with the International Classification of Functioning (ICF) model of disability (21).

Our study has limitations. Given that we used cross-sectional survey data, we are aware that the causal inferences of our findings are limited. The measurement for obtaining disability-related information within NHIS also has its weaknesses. The cases of people with lifelong disabilities are not prevalence estimates (19). These people first had to identify that they had a limitation. Thus, we are missing persons with disabilities who did not identify their disability status as a limiting factor in their lives. Furthermore, our coding of lifelong disability onset was based on respondent recall as to how long they had a limitation. Given that these reports resulted in some people being classified as having lifelong old-age problems or pregnancy problems, the methods are not perfect. However, in the absence of long-term cohort data for people with disabilities, retrospective reports provide a valuable window into the timing of disability onset in relation to development of chronic conditions.

A common limitation in disability epidemiology is having a limited sample size (22). Although NHIS oversamples certain populations (ie, some races and older adults), it does not oversample people with disabilities. As a result, the sampling methods make it difficult to randomly select disability populations. This is particularly true of disability subpopulations that are considered rare. Therefore, despite combining 7 years of data, the sample of adults with lifelong disabilities was still small compared with the sample without limitations. When examining subpopulations of people with disabilities, the sample reduced further, thus providing limited power with wide confidence intervals for subsample analyses. That even these wide confidence intervals did not overlap suggests that the disparities are substantial. However, some of the significant differences we found may have been an artifact of the inflated size of the comparison group relative to those with lifelong disabilities. Future analyses with matched cases would be helpful in addressing this concern. We were also limited in our ability to control for other factors associated with chronic conditions (eg, income, education), because these variables were not included in the Sample Adult file. With the exception of the variable on work status (ie, employed during the past year or not), our analyses were unable to determine the extent to which the chronic conditions found among people with lifelong disability were associated with lower socioeconomic status.

Despite these limitations, the NHIS has great utility as a disability data source. A major strength is that NHIS data provide information surrounding the type and onset of limitation. This information is often not available in surveillance data, particularly when disability is measured by a broad definition that clusters together various subpopulations of disability (22).

According to the Centers for Disease Control and Prevention, the health conditions we examined are among the most common, costly, and preventable health problems in the United States (20). Our findings indicate that people with lifelong disabilities had increased odds of developing these conditions compared with those without limitations. Although some activity limitations may develop subsequent to 1 or more chronic conditions, our findings suggest that activity limitation is a potential risk factor for initially developing chronic conditions. These findings support the urgency of implementing health education, disease prevention, and health-promotion programming for disability populations across the lifespan. Health care professionals should provide additional follow-up examinations for disability populations to identify risk factors related to chronic conditions and reduce further health disparity.

Public health and health care professionals should also recognize that people with disabilities experience the same common health conditions and at even greater frequency than those without limitations. Thus, acknowledging that both disability and chronic conditions may concurrently be present is important when addressing the interaction between disability and poor health, regardless of which came first.
